# *GmGBP1*, a homolog of human *ski* interacting protein in soybean, regulates flowering and stress tolerance in Arabidopsis

**DOI:** 10.1186/1471-2229-13-21

**Published:** 2013-02-06

**Authors:** Yanwei Zhang, Lin Zhao, Haiyan Li, Yang Gao, Yongguang Li, Xiaoxia Wu, Weili Teng, Yingpeng Han, Xue Zhao, Wenbin Li

**Affiliations:** 1 Key Laboratory of Soybean Biology in Chinese Education Ministry, College of Agronomy, Northeast Agricultural University, Harbin, 150030, China

**Keywords:** *GmGBP1*, Abiotic stress, Flowering, Day-length, Gibberellin

## Abstract

**Background:**

*SKIP* is a transcription cofactor in many eukaryotes. It can regulate plant stress tolerance in rice and Arabidopsis. But the homolog of SKIP protein in soybean has been not reported up to now.

**Results:**

In this study, the expression patterns of *soybean GAMYB* binding protein gene (*GmGBP1*) encoding a homolog of *SKIP* protein were analyzed in soybean under abiotic stresses and different day lengths. The expression of *GmGBP1* was induced by polyethyleneglycol 6000, NaCl, gibberellin, abscisic acid and heat stress. *GmGBP1* had transcriptional activity in C-terminal. *GmGBP1* could interact with R2R3 domain of *GmGAMYB1* in SKIP domain to take part in gibberellin flowering pathway. In long-day (16 h-light) condition, transgenic Arabidopsis with the ectopic overexpression of *GmGBP1* exhibited earlier flowering and less number of rosette leaves; Suppression of *AtSKIP* in Arabidopsis resulted in growth arrest, flowering delay and down-regulation of many flowering-related genes (*CONSTANS*, *FLOWERING LOCUS T*, *LEAFY*); Arabidopsis *myb33* mutant plants with ectopic overexpression of *GmGBP1* showed the same flowering phenotype with wild type. In short-day (8 h-light) condition, transgenic Arabidopsis plants with *GmGBP1* flowered later and showed a higher level of *FLOWERING LOCUS C* compared with wild type. When treated with abiotic stresses, transgenic Arabidopsis with the ectopic overexpression of *GmGBP1* enhanced the tolerances to heat and drought stresses but reduced the tolerance to high salinity, and affected the expressions of several stress-related genes.

**Conclusions:**

In Arabidopsis, *GmGBP1* might positively regulate the flowering time by affecting *CONSTANS*, *FLOWERING LOCUS T*, *LEAFY* and *GAMYB* directly or indirectly in photoperiodic and gibberellin pathways in LDs, but *GmGBP1* might represse flowering by affecting *FLOWERING LOCUS C* and *SHORT VEGETATIVE PHASE* in autonomous pathway in SDs. *GmGBP1* might regulate the activity of ROS-eliminating to improve the resistance to heat and drought but reduce the high-salinity tolerance.

## Background

Flowering time plays key roles in plant development, plant adaptation to growing regions [1], crop yield [2,3] and disease resistance [4]. Abiotic stress such as drought, high salinity and heat, is another major limiting factor for crop yield [2]. Genetic and molecular analyses has revealed that several distinct but linked signaling pathways regulate the flowering-time in response to light and temperature signals or to internal signals such as vernalization, autonomous pathways and gibberellin signal pathway [1,5-7]. Interaction of different signal transduction pathways before their convergence may allow a coordinated regulation of the activity of the respective pathways. The signals from these pathways are integrated through up-regulating the expression of one or more common target genes: *FT*, *SOC1* and *LFY* to determine when flowering. The expressions of *SOC1* and *FT* are negatively regulated by *FLC* but positively regulated by *CO*[8,9]. *FLC*, a MADS-box transcription factor, is a negative regulator of floral initiation and an integrator of the autonomous and vernalization pathways. *CO*, encoding a B-box zinc-finger transcription factor, integrate circadian clock and light signals and up-regulate the expression of *FT* and *SOC1* directly in the long-day pathway [10]. *CONSTANS* and *GAMYB* (*AtMYB33*) regulate the expression of LFY by binding with different cis-acting elements in the promoter of LFY respectively [11-14]. *SKIP* is a transcription cofactor in many eukaryotes. All the *SKIP* homologs identified so far contained a SKIP domain with an S-N-W-K-N peptide signature and may have conserved basic functions, such as acting as a cofactor in transcription and splicing [15]. However, the derived or additional functions of the *SKIP* homologs varied among species. *SKIP* was an essential protein for pre-mRNA splicing in *Saccharomyces cerevisiae*[16]. In *Drosophila melanogaster*, *SKIP* was involved in ecdysone-stimulated transcription [17] and known to be a coactivator in Notch [18,19]. In *Caenorhabditis elegans*, *SKIP* was an essential component of many RNA polymerase II transcription complexes and indispensable for *C*. *elegans* development [20]. In *Hordeum vulgare L*., *SKIP* could interact with *GAMYB*[21]. In *Oryza sativa L*., *OsSKIPa* could positively modulate cell viability and stress tolerance [22]. *AtSKIP* functions as not only a positive regulator and putative potential transcription factor in the abiotic stress signaling pathway in Arabidopsis [23], but also a component of the spliceosome linking alternative splicing and the circadian clock in Arabidopsis [24]. However, there has been no report on the identification of *SKIP* homologs in soybean and on their functions in the regulation of flowering time in any species so far.

In this study, the expression patterns of soybean *GAMYB* binding protein encoding a homolog of *SKIP* protein were analyzed under several abiotic stresses and light conditions. Yeast two-hybrid assay was performed to identify the interactions between *GmGBP1* and *GmGAMYB1*. The phenotypes of transgenic Arabidopsis lines in different stress and day-length were analyzed to study the function of *GmGBP1*.

## Results

### *GmGBP1* encodes a homolog of the human SKIP transcriptional coregulator

The SKIP homolog gene in soybean was obtained from subtracting long-day from short-day treated mRNA. The predicted gene, named as *GmGBP1* (*Glycine max GAMYB*-binding protein gene, GenBank DQ112540), wasamplified from the cDNA of DongNong 42 using PCR. The full length cDNA of *GmGBP1*, containing 2253 bp with an open reading frame of 1,839 bp, was predicted to encode 612 amino acids. *GmGBP1* protein had a predicted isoelectric point (pI) of 8.69 and a molecular weight (MW) of 69.10 kDa.

**Figure 1:**
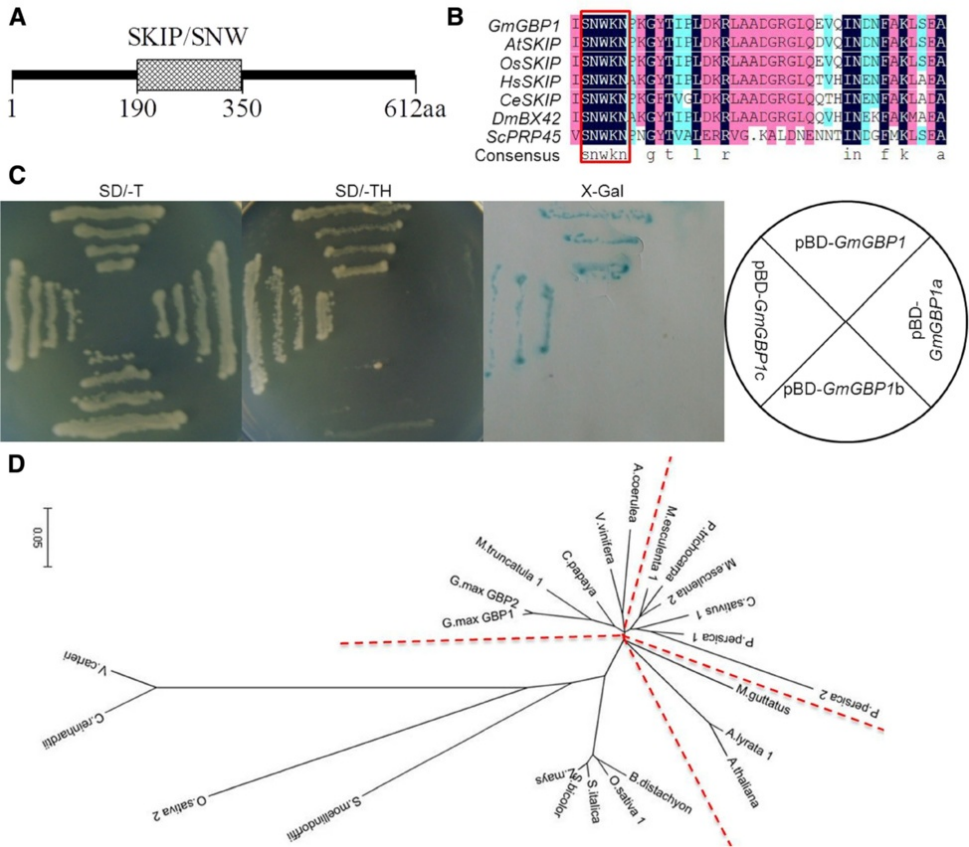
**Identification of *SKIP *homologs in soybean.** (**A**) Predicted domains and motifs of soybean *SKIP* homolog, *GmGBP1*. (**B**) Multiple alignment of the predicted amino acid sequences in peptide signature of *SKIP* homologs from soybean (*GmGBP1*, DQ112540), Arabidopsis (*AtSKIP*, O80653), rice (*OsSKIP*,), human (*HsSKIP*, Q13573), *Caenorhaabditis elegans* (*CeSKIP*, Q22836), *Drosophila* (*DmBX42*, P39736), *Saccharomyces cerevisiae* (*ScPRP45*, Q09882). (**C**) Detection for self-transactivation of *GmGBP1*. The deletion mutants (shown on right), *GmGBP1*, *GmGBP1a* (amino acids 1–189), *GmGBP1*b (amino acids 190–356) and *GmGBP1*c (amino acids 357–612), were constructed by PCR. Self-transactivation, as illustrated by the color reaction in the 5-bromo-4-chloro-3-indolyl-β-D-galactopyranoside(X-Gal) assay, occurred with 1h for pBD-Gm*GBP1* and pBD-Gm*GBP1*c constructs. Overnight incubation failed to produce a self-activation reaction with the pBD-*GmGBP1*a and pBD-*GmGBP1*b constructs. And the transformants were streaked on SD agar plates lacking His, Trp and incubated at 30°C for 72 h. The pBD-*GmGBP1* and pBD-*GmGBP1*c constructs could grow normally but no growth for the pBD-*GmGBP1*a and pBD-*GmGBP1*b constructs. (**D**) Phylogenetic relationships of *SKIP* homologs in plant constructed using the neighbor-joining method with the program MEGA 5.05.

Sequence alignment indicated that *SKIP* proteins were highly conserved in eukaryotes. The SKIP protein domain of *GmGBP1* was located between amino acids 190 and 360 (Figure 1A) with an S-N-W-K-N peptide signature (Figure 1B). A BLAST search was performed against the assembled soybean genome databases (http://www.phytozome.org) to analyze the *SKIP* sequences in viridiplantae. The phylogenetic tree based on the *SKIP* sequences agreed well with the evolutionary relation among these species those were divided into four groups (Figure 1D).

A yeast transformation was used to detect the transcriptional activity of *GmGBP1*. *GmGBP1*, *GmGBP1a*, *GmGBP1b* and *GmGBP1c* were fused to the *GAL4* DNA-binding domain and transformed into yeast strain *YRG*-*2* cells respectively. The proteins of *GmGBP1* and *GmGBP1c* were both capable of inducing *Lac*Z expression in yeast cells and could grow in the medium lacking His, indicating that *GmGBP1* protein had transcriptional activity in its C-terminal domain (Figure 1C).

### *GmGBP1* expression in soybean

**Figure 2:**
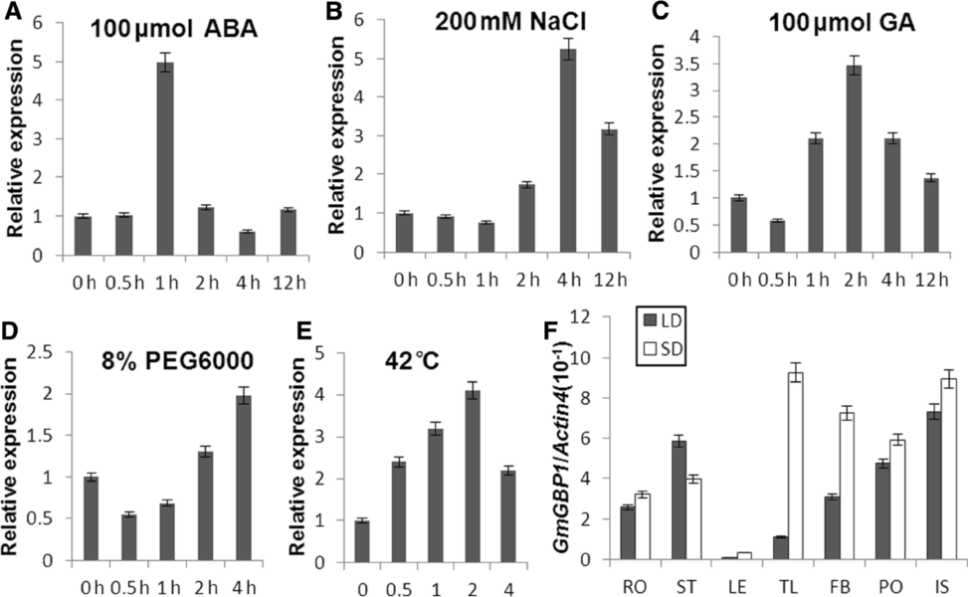
**Expression patterns of *GmGBP1 *in soybean.** (**A**, **B**, **C**, **D** and **E**) Expression level of *GmGBP1* in leaf of soybean treated with 100μmol ABA, 200mM NaCl, 100μmol GA, 8% PEG6000 and heat stress respectively. (**F**) Tissue-specific expression of *GmGBP1* in LDs and SDs. Tissues tested were root (RO), stem (ST), leaf (LE), trifoliate leaf (TL), flower bud (FB), pod (PO) and immature seed (IS).

A quantitative RT-PCR was performed to analyze the expression of *GmGBP1* in soybean. *GmGBP1* could express in all tissues or organs investigated in both LDs and SDs, although the expression levels were different. The expression levels in stem and immature seed were much higher than those in root, trifoliate leaf, flower bud, pod and leaf. The trifoliate leaf had the lowest expression level in LDs, but the highest expression level in SDs in the seven samples detected. All the organs, except of stem, showed higher expression in SDs than in LDs (Figure 2F). The expression of *SKIP* homologs in rice and Arabidopsis have been confirmed to be affected by stresses and phytohormones (Xin et al. 2009; Gah-Hyun et al. 2010). Therefore, the expression level of *GmGBP1* in soybean was also analyzed with stresses and phytohormones treatments in the present study. The results indicated that the transcriptional level of *GmGBP1* was increased after treated with 100 μmol ABA (Figure 2A), 100 μmol GA (Figure 2C), 200 mM NaCl (Figure 2B), 8% PEG6000 (Figure 2D) and temperature 42°C respectively (Figure 2E).

### *GmGBP1* interacts with *GmGAMYB1* in yeast cells

**Figure 3:**
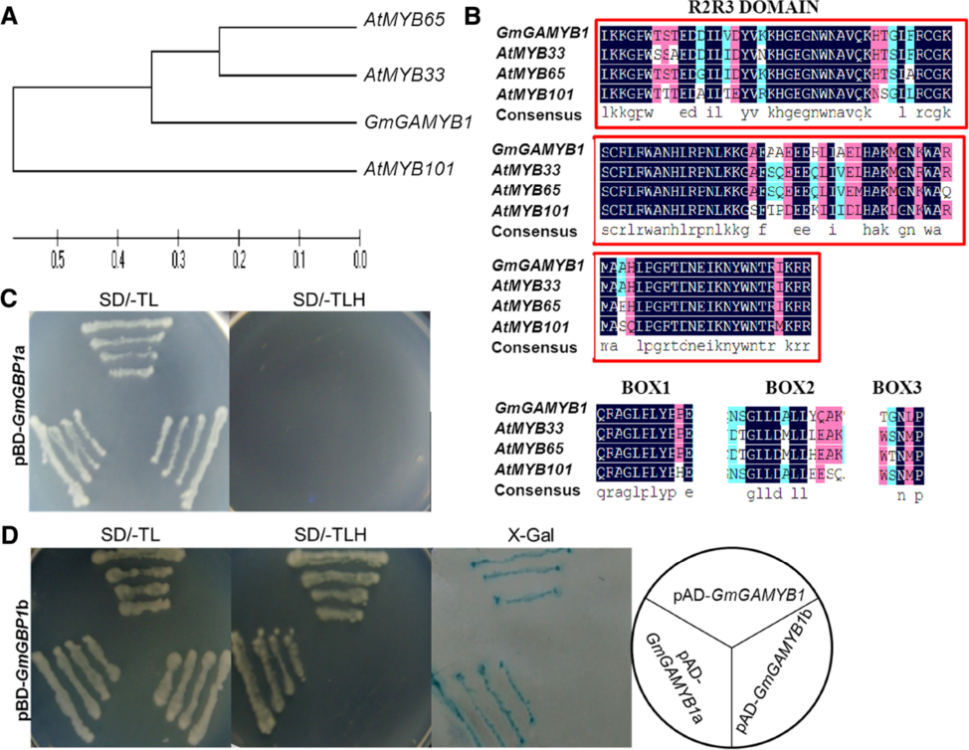
**Yeast Two**-**Hybrid Assay of Interaction between *GmGBP1 *and *GmGAMYB1.*** (**A**) Phylogenetic relationships between *GmGAMYB1* (HM447241) and *GAMYB* homologs (*AtMYB33*, NM_180448; *AtMYB65*, NM_111977; *AtMYB101*, NM_128805.3) in Arabidopsis using the neighbor-joining method with the program MEGA 5.05. (**B**) Multiple alignment of the amino acid sequences in domain and motify of *GmGAMYB1*, *AtMYB33*, *AtMYB65* and *AtMYB101*. (**C**) The growth of Yeast *YRG*-*2* cells cotransformated by pBD-GmGBP1a with pAD-*GmGAMYB1*, pAD-*GmGAMYB1*a (amino acids 1–141) or pAD-*GmGAMYB1*b (amino acids 142–538) on synthetic minimal (SD) medium lack of Trp and Leu (SD/-TL) or Trp, Leu and His (SD/-TLH). (**D**) The growth of Yeast YRG-2 cells cotransformated by pBD-*GmGBP1*b with pAD-*GmGAMYB1*, pAD-*GmGAMYB1*a or pAD-*GmGAMYB1*b on SD/-TL and SD/-TLH, and the X-Gal assay for the transformations.

In barley, it was confirmed that *HvGAMYB* could interact with a SKIP domain by yeast two-hybrid system [21]. Therefore, a yeast two-hybrid assay was performed to study the interaction between *GmGBP1* and *GmGAMYB1*. *GmGAMYB1* contained a typical R2/R3-*MYB* DNA-binding domain and three conserved regions (BOX1, BOX2 and BOX3). *GmGAMYB1* was more homologous to *AtMYB33* rather than *AtMYB65* and *AtMYB101* (Figure 3A, Figure 3B). As *GmGBP1* showed transcriptional activity in the C-terminal domain, the pBD-*GmGBP1*a and pBD-*GmGBP1*b were chosen for the bait vectors to search the protein interaction with *GmGAMYB1*. Moreover, the prey was divided into two parts (1–141,142-538) by the domain area of R2R3, as pAD-*GmGAMYB1*, pAD-*GmGAMYB1*a and pAD-*GmGAMYB1*b. The co-transformation with pBD-*GmGBP1*a and the prey plasmid could not grow on SD/-TLH, indicating that the section of *GmGBP1* had no ability to interact with *GmGAMYB1* (Figure 3C). Furthermore, the yeast cells with pBD-*GmGBP1*b and pAD-*GmGAMYB1*b showed no ability to interact with *GmGAMYB1* (Figure 3D). In contrast, the section b of *GmGBP1* interacted with *GmGAMYB1* by binding to the domain of *GmGAMYB1* as the result of the growth on SD/-TLH, exhibiting the positive color reaction (blue on the X-Gal filter assay, Figure 3B). 

### The homozygous mutant *atskip* of *Arabidopsis* was lethal at late stage

**Figure 4:**
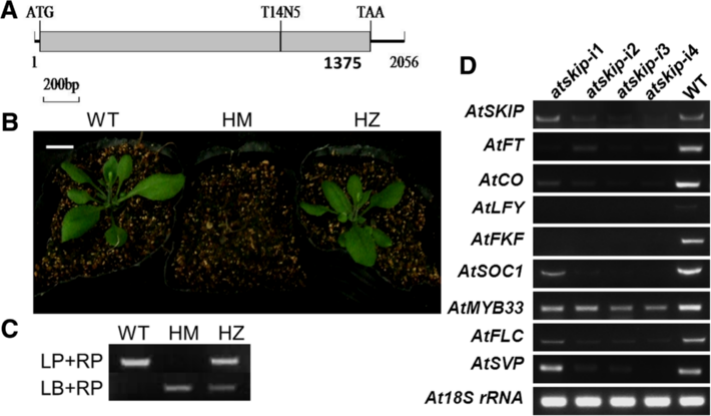
**Identification of *atskip *mutant plants in phenotypes and genotype.** (**A**) A T-DNA insertion caused the mutant of *AtSKIP*. (**B**) Phenotypes of homozygous atskip mutant (HM), heterozygous *atskip* mutant (HZ) and wild type Col-0 plant (WT). Bars = 1.0cm. (**C**) PCR tests for genotype to identify the mutant. (**D**) RT-PCR analysis for the down-regulated genes (*AtFT*, NM_105222.2; *AtCO*, NM_001036810.1; *AtLFY*, NM_125579; *AtFKF*, Q9C9W9; *AtSOC1*, NP_182090.1; *AtFLC*, Q9S7Q7; *AtSVP*, Q9FUC1) in *atskip*-i plants.

An insertion of a T14N5 fragment at the 1344 bp of exon of *AtSKIP* caused mutant (Figure 4A). The mutant *atskip* had the same germination rate with the WT plant (>99%), and all of the plants could grow normally in the MS medium. However, 100% of the homologous plants died within 2 weeks after transplanting in soil before flowering whereas all WT and heterozygous plants grew normal under the same conditions (Figure 4B, Figure 4C). The homozygosity of mutant was detected by PCR and RT-PCR after transplanting in soil.

Considering the homozygous mutant of *AtSKIP* was lethal, RNAi was used to produce the *atskip*-i plant to suppress the expression of this gene. Although the expression of *AtSKIP* was only partially suppressed (Figure 4E), the plants showed abnormal phenotypes such as late flowering, small rosette leaves. According to RT-PCR analyses, the expressions of several key flowering-related genes were down-regulated (Figure 4E). The promotion and inhibitory factors of flowering were both down-regulated, which suggested that *SKIP* might be involved in more than two different flowering pathways.

### Ectopic expression of *GmGBP1* alters stress tolerance in Arabidopsis

**Figure 5:**
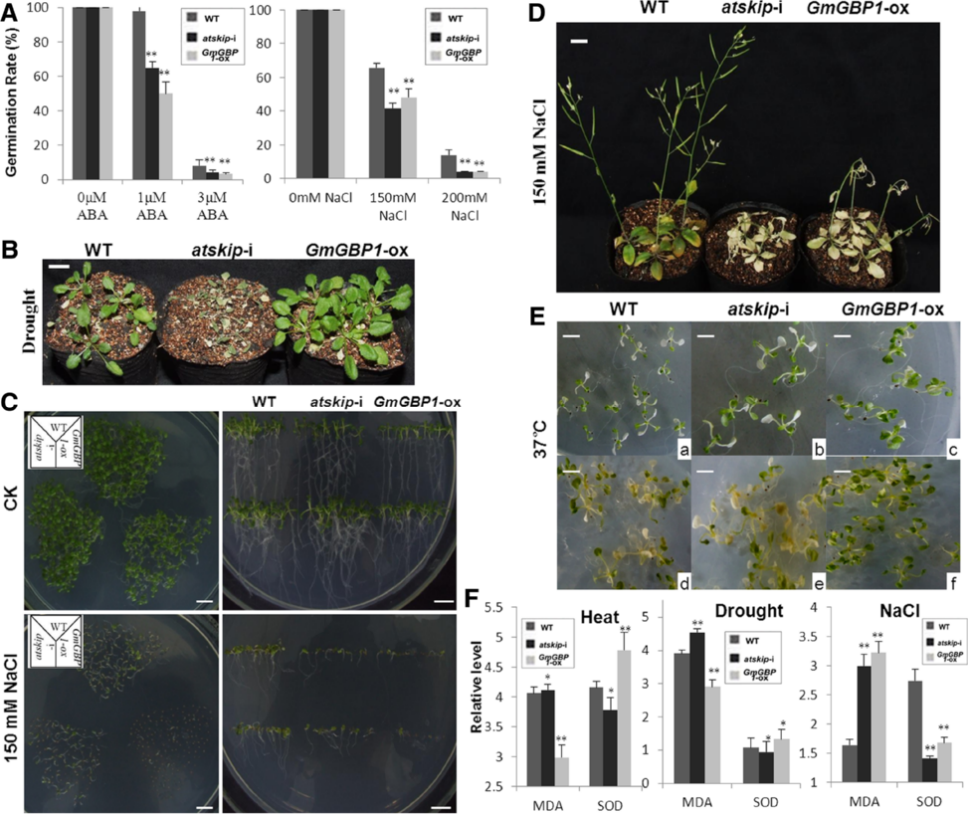
**Performance of seedling under stress condition.** (**A**) The germination rate of plants treated with 1 μM ABA, 3 μM ABA, 150 mM NaCl or 200 mM NaCl individually. (**B**) Performance of seedlings treated with drought. Effect of 20 days old plants growing in soil in SDs treated with drought (no water for 7days), photos at 3 days after rewatering, Bars = 1.0 cm. (**C**) Phenotypes of seedlings treated with 150mM NaCl. Germination of plants growing in MS medium with 150mM NaCl, photos at 7 days after germination; Growth of plants cultivated in MS medium with 150mM NaCl vertically, photos at 10 days after germination. Bars = 1.0 cm. (**D**) Effect of 20 days old plants growing in soil in LDs treated with 200mM NaCl every other day for 3 times, photos at 10 days after treated. Bars = 1.0 cm. (**E**) Performance of seedling under heat: a, b and c: effect of plants of 7 days old under 37°C for 5 days; d, e and f: effect of plants of 15 days old under 37°C for 8 days. Bars = 0.5 cm. (**F**) The relative level of MDA and SOD of plants under stress conditions. 20 days old plants growing in soil in SDs treated with 42°C for 36h, samples were prepared from rosette leaf after recovered for 1 day; 20 days old plants growing in soil in LDs, samples were prepared from rosette leaf after treated with drought (no water for 7 days); 20 days old plants growing in soil in LDs treated with 200mM NaCl every other day for 3 times, samples were prepared from rosette leaf after 7days. Data were from more than 8 individuals for each genotype ± SD. * *t* test, P < 0.05; ** *t* test, P < 0.01.

Since *GmGBP1* was up-regulated by stresses and stress-related phytohormones, *Arabidopsis thaliana* plants, containing extra copies of *GmGBP1* gene, was produced to investigate whether overexpression of this gene could enhance stress tolerance. The transgenic lines were confirmed by PCR and RT-PCR with homolog-specific primers (data not shown). In the MS medium containing ABA (1 and 3 μM), the germination rates of *GmGBP1*-ox and *atskip*-i plants were both reduced compared to WT (Figure 5A), indicating that ectopic expression of *GmGBP1* could increase the growth-suppression effect of ABA and *AtSKIP* might have the ability of reducing the negative effect of ABA. In the MS medium containing NaCl (150 and 200 mM), the germination rates of *GmGBP1*-ox and *atskip*-i plants were also lower than the WT plants (Figure 5A, Figure 5C), suggesting that *GmGBP1* decreased salt tolerance and *AtSKIP* improved the ability to salt tolerance. However, the germination rates of the three plants under 300 mM mannitol showed no difference (data not shown). A further study was performed to detect the stress tolerance of *GmGBP1*. When 20 days old plants growing in soil under SDs were treated with drought condition (no watering for 7 days), nearly all of the *GmGBP1*-ox plants were recovered at 3 days after re-watering, whereas only 40-60% of WT plants and none of *atskip*-i plants were recovered (Figure 5B). The root lengths of both Gm*GBP1*-ox and *atskip*-i plants were shorter than that of WT plants in the MS medium treated with 150 mM NaCl for 10 days (Figure 5D); The *GmGBP1*-ox and *atskip*-i plants treated with 200 mM NaCl for 10 days under LDs were nearly dead, while 80-90% of WT plants survived (Figure 5D).

Heat tolerance experiment revealed that *GmGBP1* might improve heat tolerance of Arabidopsis plants with ectopic expression of *GmGBP1*. 7days old plants of transgenic Arabidopsis, treated with 37°C for 5 days, exhibited symptom of flagged leaves, but WT and *atskip*-i plants showed more serious symptom with whitened leaves (Figure 5E: a, b and c). Furthermore, WT and *atskip*-i plants of 15days old showed more whitened leaves, even dead after treated with 37°C for 8 days, whereas *GmGBP1*-ox plants showed more vital (Figure 5E: d, e and f).

The *GmGBP1*-ox plants had a higher Superoxide dismutase (SOD) level than both *atskip*-i and WT plants under heat or drought conditions. The malondialdehyde (MDA) level of *GmGBP1*-ox plants was much lower than both *atskip*-i and WT plants, indicating that the ectopic expression of *GmGBP1* in Arabidopsis might enhance the heat and drought tolerance. However, the highest MDA level and the lowest SOD level of *GmGBP1*-ox plants implied the reduced salt tolerance in *GmGBP1*-transgenic Arabidopsis (Figure 5F).

### Stress resistance-related genes are affected by *GmGBP1*

**Figure 6:**
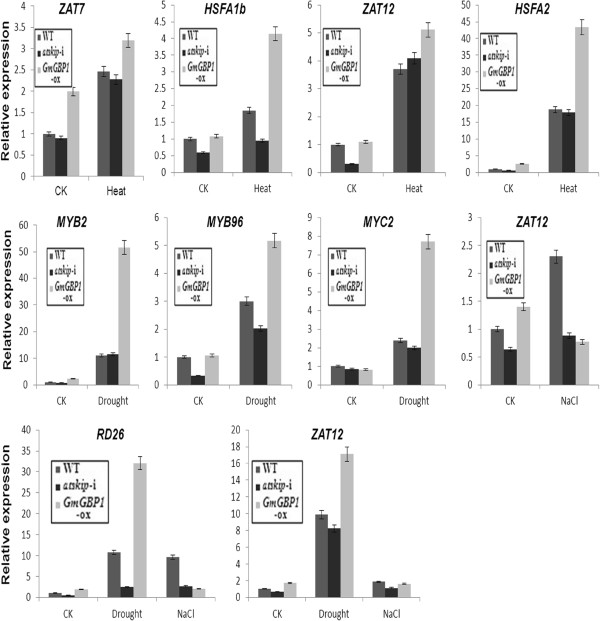
**Expression of stress resistance-related genes in WT**, ***GmGBP1***-**ox and *atskip***-**i plants.***ZAT7*, NM_114478.3; *ZAT12*, AY050915.1; *HSFA1b*, NM_121688; *HSFA2*, NM_001124916; *RD26*, NM_001084983; *MYC2*, NM_102998; *MYB96*, NM_125641; *MYB2*, NM_130287.

To gain further insight into the mechanism of the altered stress resistance of *GmGBP1*-ox plants, transcript levels of 8 stress-responsive genes [25-28] were assayed in the *GmGBP1*-ox, *atskip*-i and WT plants under normal and stress conditions. The expression levels of all the 8 genes in *GmGBP1*-ox were higher than WT and *atskip*-i plants (Figure 6). Moreover, *ZAT7*, *ZAT12*, *HSFA1b* and *HSFA2* showed significantly higher expression level in *GmGBP1*-ox than in WT and *atskip*-i plants after treated with 42C for 2h (Figure 6), *MYB2*, *MYB96*, *MYC2*, *RD26* and *ZAT12* were up-regulated after drought for 7days (Figure 6). These results suggested that overexpression of *GmGBP1* can increase the transcript levels of some stress-resistance genes under drought or heat stress conditions, thus improving the drought or heat resistance of the transgenic plants. However, the expression levels of *RD26*, *ZAT7* and *ZAT12* in *GmGBP1*-ox plants were significantly lower than WT and *atskip*-i plants after treated with 200 mM NaCl for 7 days (Figure 6), indicating that *GmGBP1* might reduce the resistance to high salinity. 

### Early flowering of *GmGBP1*-ox plants in long-day condition

**Figure 7:**
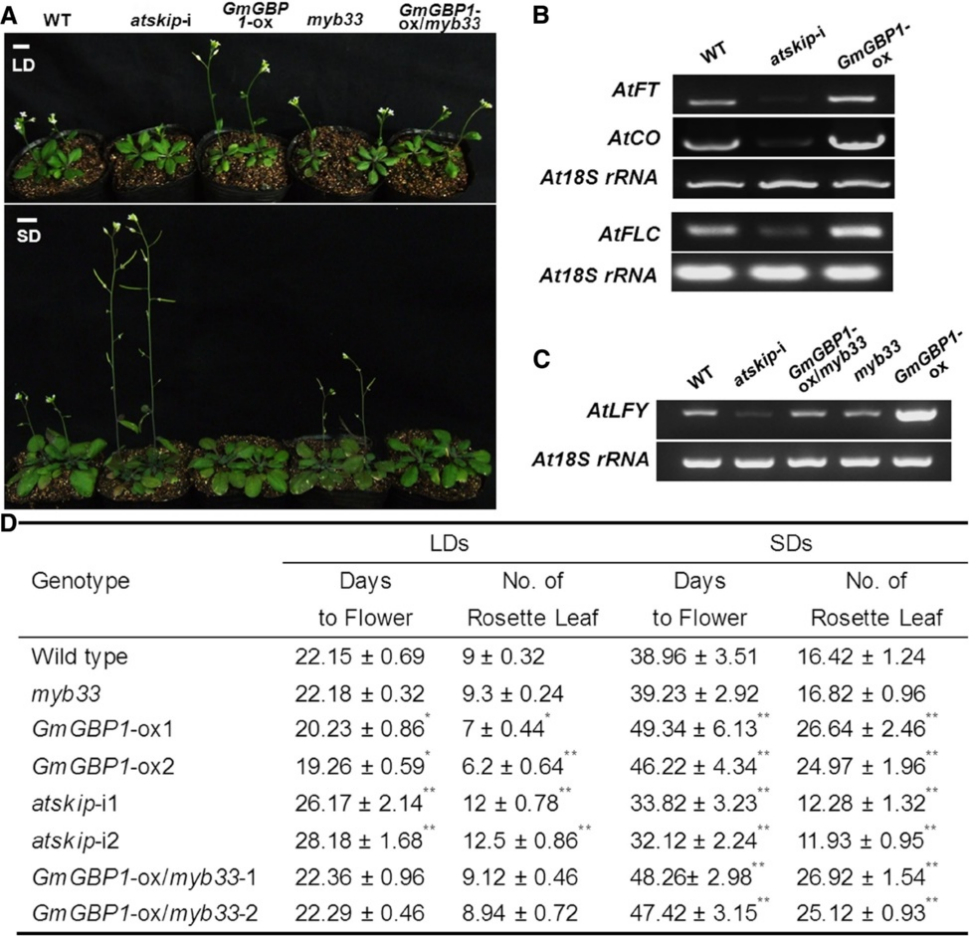
**Effect of WT**, ***atskip***-**i**, ***GmGBP1***-**ox**, ***myb33 *and *GmGBP1***-**ox**/***myb33*plants.** (**A**) Phenotypes of plants in LDs and SDs. Photos at 25 days after germination in LDs; Photos at 42 days after germination in SDs. Bars = 1cm. (**B**) Expression level of flowering related gene in Arabidopsis. RT-PCR analysis for *AtCO* and *AtFT* in WT, *atskip*-i, *GmGBP1*-ox plants in LDs; RT-PCR analysis for *AtFLC* for plants in SDs. Samples were prepared from rosette leaves before bolting in LDs or SDs. (**C**) Expression level of *AtLFY* in plants. Samples were prepared from rosette leaves before bolting in LDs. (**D**) Flowering time of plants in LDs and SDs. Flowering time was measured as the total number of rosette leaves or days to bolting. Data were from more than 20 individuals for each genotype ± SD. WT plants compared with *atskip*-i, *GmGBP1*-ox, *myb33* and *GmGBP1*-ox/*myb33* plants, respectively. * showed significant to WT plant at 0.05 level (P < 0.05) by *t*-test; ** showed significant to WT plant at 0.01 level (P < 0.01) by *t*-test.

The response to day-length implied the possibility of flowering-related functions of *GmGBP1*. In LDs condition (16-h light), the T3 plants of *GmGBP1*-ox Arabidopsis flowered 2 days earlier and had fewer rosette leaves than WT plants, and the *atskip*-i plants flowered significantly late (Figure 7A, Figure 7D). Furthermore, the expressions of *CONSTANS* (*CO*) and *FLOWERING LOCUS T* (*FT*) in *GmGBP1*-ox plants were increased compared to WT and *atskip*-i plants, and *atskip*-i plants had the lowest expression levels of *CO* and *FT* (Figure 7B), indicating that *GmGBP1* could control the flowering time by up-regulating flowering-related gene *CO* and *FT* in LDs.

However, in the short-day (SD) condition, the T3 plants of *GmGBP1*-ox Arabidopsis flowered latest and had the most rosette leaves (Figure 7A, Figure 7D). In contrast, *atskip*-i plants showed the earliest flowering and had the fewest rosette leaves. This result suggested that there might be some other ways to regulate flowering in SDs. The key flowering inhibitor gene, *FLC*, was significantly up-regulated in *GmGBP1*-ox plants (Figure 7B). All the results suggested autonomous pathway could account for the late flowering phenotype.

### *GAMYB* genetically acts downstream of *GBP* to regulate flowering time

Previous reports showed that *AtMYB33* influenced flowering by mediating GA responsiveness of the *LFY* promoter [12,14], and was down-regulated in *atskip*-i plant in this study (Figure 4D). *myb33* mutant plants (SALK058312) and *myb33* mutant plants containing extra copies of *GmGBP1* gene were introduced to investigate the impact of the interaction between *GmGBP1* and *GmGAMYB1* on flowering. In LDs, no effect on flowering time and leaf numbers was observed in *GmGBP1*-ox/*myb33* plants (Figure 7A, Figure 7D), and the expression level of *LFY* in *GmGBP1*-ox/*myb33* plants was same to the level of WT, but *GmGBP1*-ox plants had a high level of *LFY* (Figure 7C). *GmGBP1* was directly located on the upstream of *GAMYB*, and regulate flowering in LDs.

Since *GmGBP1* regulated flowering in autonomous pathway in SDs, a further study was performed to investigate the responsibility of *MYB33* in the late flowering phenotype. The result revealed that *GmGBP1*-ox/*myb33* plants also showed late flowering like *GmGBP1*-ox plants (Figure 7A and Figure 7D), suggesting that *GmGBP1* was independent of *MYB33* in delaying flowering time in SDs.

## Discussion

### Essential roles of *SKIP* for plant growth and stress tolerance

SKIP protein, as a spliceosome component, played a vital role in maintaining cell viability in yeast (*PRP45*), *C*. *elegans* (*CeSKIP*) and *O*. *sativa* (*OsSKIPa*) [16,20,22]. The homozygous mutant of *AtSKIP* in Arabidopsis resulted in death of plants, which was similar to the phenotype of severe growth arrest and even death of *O*. *sativa* with suppression of *OsSKIPa* and embryonic arrest of the *CeSKIP* mutant in *C*. *elegans*, which indicated that *AtSKIP* was also required for maintaining cell viability and normal growth in Arabidopsis. The indispensable role of *SKIP* homolog in keeping normal cell viability and growth might be conserved in plants, which was supported by that the plant *SKIP* homologs possessed the conserved SKIP domain for cell viability identified in *PRP45*. However, it needs further study to confirm whether the *SKIP* homologs in soybean could have the conserved function for cell viability.

Drought and high-salinity repressed plant growth and limited seed yield. *SKIP* homologs in Arabidopsis and rice could improve the tolerance to drought and high-salinity [22,23]. In this study, the ectopic expression of *GmGBP1* in Arabidopsis could enhance the tolerance to drought. However, *GmGBP1*-ox plants showed a salt-sensitive appearance, although *atskip*-i plants reduced the tolerance to salt as previous report [23]. Thus, the tolerance of *SKIP* homologs to salt might be not conserved in plant. Heat, one of serious environmental stresses, affected the growth of plants and the productivity of crops. There was no report about heat tolerance of *SKIP* homologs previously. Our result showed that *GmGBP1*-ox plants increased the tolerance to heat whereas *atskip*-i plants reduced the tolerance to heat, indicating that *AtSKIP* has the ability of heat tolerance.

Reactive oxygen species (ROS) could be induced by abiotic stresses, and over-accumulation of ROS could lead to cell damage and even death. SOD was used to eliminate ROS, and MDA was the intermediate product during the elimination of ROS [29,30]. In this study, the raised content of ROS in *GmGBP1*-ox plants treated with salinity and the decreased content of ROS in *GmGBP1*-ox plants treated with heat (or drought) indicated that the altered stress tolerance of *GmGBP1*-ox plants may be partially due to the regulation of the activity of ROS-eliminating.

### *GmGBP1* regulates flowering time

Interestingly, in this study ectopic expression of *GmGBP1* in Arabidopsis induced earlier flowering in LDs and late flowering in SDs. The finding of a flowering-regulation function from a *SKIP* homolog has not yet been reported in any plants so far. The specific function of *GmGBP1* in regulating flowering time might be especially useful for developing soybean cultivars with the adaptability to broad grow regions.

Flowering time was controlled by several signaling pathways, such as the day-length, vernalization, autonomous pathways and gibberellin signal pathway [1,5-7], and it could be measured by scoring the number of rosette leaves at flowering time and the number of days from germination to bolting in Arabidopsis [31]. The number of rosette leaves at flowering time and the number of days from germination to bolting measured in *GmGBP1*-ox plants were both reduced in LDs, suggesting that ectopic expression of *GmGBP1* induced early flowering. *atskip*-i plants delayed flowering in LDs supported the early flowering of *GmGBP1*-ox plants in another hand. However, when *GmGBP1* ectopicly expressed in *myb33* plants, no early flowering could be observed in LDs, indicating that the early flowering function of *GmGBP1* might depend on the existence of *MYB33*. When the transgenic lines were transferred into SDs, the phenotypes of flowering were all changed. The flowering times of *GmGBP1*-ox and *GmGBP1*-ox/*myb33* plants were both delayed significantly, and *atskip*-i displayed the early flowering phenotype in SDs.

The expression levels of numerous flowering-related genes could be induced by day-length or gibberellin. The expression level of *GmGBP1* was regulated by both day-length and gibberellin, suggesting that *GmGBP1* might participate in both day-length and gibberellin signal pathways. In *atskipi* plants of Arabidopsis, the expression levels of more than 10 flowering-related genes were affected. In particular, the mRNA levels of several flowering integrators (*FTLFY* and *SOC1*) were significantly lower. Our results showed that *FT* and *LFY* were both up-regulated in *GmGBP1*-ox plants in LDs, but *LFY* showed no change when *GmGBP1* was ectopic expression in *myb33* mutants. Considering the interaction between *GmGBP1* and *GmGAMYB1*, and the conserved function on photoperiodic flowering of FT homologs in soybean [32-34], *GmGBP1* might regulate flowering time in two ways by its function as a transcription factor and interaction in LDs. On one hand, *GmGBP1* could regulate the expressions of flowering-related genes in day-length signal pathway, and on the other hand, *GmGBP1* could bind with *GmGAMYB1* in gibberellin signal pathway to control flowering time.

Many reports had revealed that GA pathway played a key role in flowering under SD condition when other regulatory pathways that promoted flowering were not active [1,7,35-37]. However, the late flowering of *GmGBP1*-ox plants indicated that there might be another way to control flowering in SDs. *FLC* was a negative regulator of floral initiation and an integrator of the autonomous and vernalization pathways. *FLC* could directly down-regulated *FT* and *SOC1* to repress the flowering in these pathways [8,9,38]. *SVP*, a MADS box transcription factor, could interact with *FLC* and acted as partially redundant repressors of flowering time with *FLC*[39]. The negative action on the phenotypes of the down-regulated *FLC* and *SVP* in *atskip*-i plants in LDs suggested that flowering time was regulated crossly by several signal pathways and *SKIP* might take part in more than two ways. In SDs, the expression of *FLC* was up-regulated in *GmGBP1*-ox plants, and was down-regulated in *atskip*-i plants as that in LDs. The late flowering of *GmGBP1*-ox plants in SDs elucidated that *GmGBP1* might delay flowering time in SDs through autonomous pathway by improving the expression level of *FLC*, a key flowering inhibitor factor.

In general, *GmGBP1* might regulate flowering time by three signal pathways. *GmGBP1* positively controlled the flowering time by regulating *CO*, *FT*, *LFY* and *GAMYB* directly or indirectly in photoperiodic and gibberellin pathways in LDs, *GmGBP1* repressed flowering by regulating *FLC* and *SVP* in autonomous pathway in SDs.

### Diverse functions of *SKIP* homologs in plant

*SKIP* had a conserved SKIP domain with an S-N-W-K-N peptide signature, and was considered as a cofactor for transcription regulation in all eukaryotes so far. However, the derived or additional functions of the *SKIP* homologs varied among species. Transgenic rice that overexpressed *OsSKIPa* exhibited stress tolerances (abscisic acid, salt, mannitol) at both seedling and reproductive stages [22]. Overexpression of the *AtSKIP* gene in Arabidopsis modulated the induction of salt tolerance, dehydration resistance and insensitivity towards abscisic acid under stress conditions [23]; However, ectopic expression of *GmGBP1* in Arabidopsis reduced tolerance to NaCl, but increased tolerance to drought and heat in this study.

Suppression of *OsSKIP* resulted in growth arrest of rice due to the reduced cell viability in the active growth regions [22]. A decrease in *AtSKIP* expression led to altered plant development with the phenotype of reduced inflorescence stems and smaller rosette leaves [23]. In this study, the homozygous *atskip* mutant was lethal to the growth, and the knockdown of *AtSKIP* showed late flowering and increased number of rosette leaves. The ectopic expression of *GmGBP1* induced premature flowering of Arabidopsis in LDs. All the study on *SKIP* homologs in plant revealed that *SKIPs* might play a vital role in the growth and development of plant, but have different function on the stress tolerance and flowering in plant.

Plant *SKIPs* were divided into four groups based on the *SKIP* sequences by phylogenetic tree (Figure 1D). The different groups among *OsSKIP*, *AtSKIP* and *GmGBP1* indicated the diversity of their functions. *SKIP* could be induced by various abiotic stresses, phytohormones treatments and day-length, but the expression patterns of *SKIP* varied among rice, Arabidopsis and soybean [22,23]. The transcriptional levels of *OsSKIP*, *AtSKIP* and *GmGBP1* were all up-regulated by ABA, NaCl and drought (PEG6000 or mannitol), but had different scales. *GmGBP1* could be induced by day-length and heat, but no related report for *OsSKIP* and *AtSKIP* in rice and Arabidopsis. The various functions of *SKIP* homologs might be also owing to the diversification of *SKIP*-interacting proteins *SIP*s. Previous studies showed that the *SKIP* homologs, *PRP45* (yeast), *BX42* (*Drosophila*), *CeSKIP* (*C*. *elegans*), *HvSKIP* (barely) and *OsSKIP* (rice) had 34, 13, 5, 1 and 35 interacted proteins, respectively [16,20-22,40,41]. Nevertheless, few *SIP*s could match each other among species. For example, both *HvSKIP* and *GmGBP1* interacted with *GAMYB* but *OsGAMYB* was not included in the 35 *OsSIP*s [21,22]. All the data indicated that *SKIP* might participate in distinct functions through the interaction with diverse proteins.

## Conclusions

In this study, *GmGBP1*,as a homolog of *SKIP* in soybean, not only regulates plant flowering time but also alters plant resistance to abiotic stress in Arabidopsis. Such a flowering study about SKIP homolog in plant has no report so far. Although both *OsSKIP* and AtSKIP could improve plant tolerance to high salinity and drought [22,23], *GmGBP1* might improve plant tolerance to heat and drought, but reduce the resistance to high salinity. The flowering and stress related functions of *GmGBP1* might be used to develop soybean cultivars with the adaptability to broad grow regions and environment.

## Methods

### Plant material, growth conditions and chemical treatments

Pure seeds of soybean cultivar ‘DongNong 42’ (photoperiod-sensitive) were obtained from Soybean Research Institute of Northeast Agricultural University (Harbin, China). To analysis the tissue-specific expression of *GmGBP1*,the root, stem, leaf, trifoliate leaves, flower bud, pod and immature seed from soybean under both LDs and SDs were sampled. To analysis the expression of *GmGBP1* under abiotic stress conditions, the soybean plants, with the first fully expanding trifoliate leaves, were transplanted into 1/4 Murashige and Skoog (MS) [42] liquid medium (no agar supplement) supplemented with 100 μmol gibberellin, 100 μmol ABA, 200 mM NaCl or 8% PEG6000. The plants that were transplanted into 1/4 MS liquid medium without the supplements of agar, gibberellin, ABA or NaCl were used as the control. The plants were sampled at 0, 0.5, 1, 2, 4 and 12 hours after transfer.

*Arabidopsis thaliana* ecotype Columbia (Col-0) was used in this study. Seeds of *atmyb33* T-DNA insertion line (Salk_058312) and *atskip* T-DNA insertion line (SAIL_681_H11) were obtained from The Arabidopsis Information Resource (TAIR).

### Identification of mutant

PCR with the gene-specific primer pair and T-DNA-specific primer were used for genotyping to identify homozygous T-DNA inserted plants. The left genomic primer (LP), right genomic primer (RP) and the left T-DNA border primer (LB) for *atskip* were as follows: LP: 5^′^ CAAGCACAAGAGAGTCCCAAG 3^′^ RP: 5^′^ CGCCACTTGCTCTCATAGTTC 3^′^ LB: 5^′^ GCCTTTTCAGAAATGGATAAATAGCCTTGCTTCC 3^′^. The LP, RP and LB primers for *myb33* were as follows: LP: 5’ ATCCAGAACTGTCAGACGCTG 3^′^ RP: 5^′^ AATTGCGTATTTGGTTGGATG 3^′^ LB: 5^′^ ATTTTGCCGATTTCGGAAC 3^′^. After confirmation of homozygous T-DNA insertion, gene knock-out was confirmed by RT-PCR with gene-specific primer.

### Gene expression analyses

Total RNA was isolated from soybean leaves using TRIzol reagent (Invitrogen, USA). Real-time quantitative RT-PCR was performed on a Chromo4 Real-Time PCR System (Bio-Rad, USA) using SYBR Green PCR Master Mix Reagent (Takara, Japan). The measured C_t_ values were converted to relative expression level using the ΔΔC_t_ method. Soybean *Actin4* gene was used as the endogenous control. Primers used were q*GmGBP1*-F and q*GmGBP1*-R; q*GmACTIN4*-F and q*GmACTIN4*-R (Table 1).

**Table 1:** Primers used in this study

**Primer names**	**Primer sequences** (**5**’–**3**’)
c*GmGBP1*-F	5’-TCCCAAAATCACCATCTA-3’
c*GmGBP1*-R	5’-TTGTAACATCCATAAGCAGT-3’
*GmGBP1*-i-F	5’-GGGGACAAGTTTGTACAAAAAAGCAGGCTGCTATGGGGAAGAGGAGTAA-3’
*GmGBP1*-i-R	5’-GGGGACCACTTTGTACAAGAAAGCTGGGTCATTACCATCCCGCATAG-3’
y*GmGBP1*-F	5’-GAATTCATGGCCACTCTGAAAGAGCTTCTTC-3’
y*GmGBP1*-R	5’-GCGGCCGCCTAATGCCCTCTTTCAAATCCAATG-3’
y*GmGBP1*a-F	5’-GAATTCATGGCCACTCTGAAAGAGCTTCTTC-3’
y*GmGBP1*a-R	5’-GCGGCCGCCTAATGCCCTCTTTCAAATCCAATG-3’
y*GmGBP1*b-F	5’-GAATTCGCGAAGTATATAAAGTACAAACCC-3’
y*GmGBP1*b-R	5’-GCGGCCGCAATTCTCTCCCCTCCAATTCTTTCA-3’
y*GmGBP1*c-F	5’-GAATTCGGGGTTGTACCAGCCGCGCCACCAG-3’
y*GmGBP1*c-R	5’-GCGGCCGCCTAATGCCCTCTTTCAAATCCAATG-3’
y*GmGAMYB1*-F	5’-GGATCCATGAGACGAATGAAGAAAGATATTG-3’
y*GmGAMYB1*-R	5’-GAATTCTTAGAGGGGCTGGAATGGATTTTCA-3’
y*GmGAMYB1*a-F	5’-GGATCCATGAGACGAATGAAGAAAGATATTG-3’
y*GmGAMYB1*a-R	5’-GAATTCCTTTTGATCCGGGTGTTCCAGTAGT-3’
y*GmGAMYB1*b-F	5’-GGATCCAACGGGCTGGGTTGCCACTTTATCC-3’
y*GmGAMYB1*b-R	5’-GAATTCTTAGAGGGGCTGGAATGGATTTTCA-3’
q*GmGBP1*-F	5’-TTTGTGAAGGAGAGTAGGGAGGAG-3’
q*GmGBP1*-R	5’-TTAGTAGAGGCCATACCAAGAGCA-3’
q*GmACTIN4*-F	5’-GTGTCAGCCATACTGTCCCCATTT-3’
q*GmACTIN4*-R	5’-GTTTCAAGCTCTTGCTCGTAATCA-3’
*AtSKIP*-F	5’-GGCTGTTTCAATGCGTTCC-3’
*AtSKIP*-R	5’-TTGGGTCAACACTTTCACTCCTA-3’
*AtLFY*-F	5’-TGTGAACATCGCTTGTCGTC-3’
*AtLFY*-R	5’-TAATACCGCCAACTAAAGCC-3’
*AtCO*-F	5’-AAGGTGATAAGGATGCCAAGGAG-3’
*AtCO*-R	5’-GGAGCCATATTTGATATTGAACTGA-3’
*AtFT*-F	5’-TGGTGGAGAAGACCTCAGGAAC-3’
*AtFT*-R	5’-TGCCAAGCTGTCGAAACAATAT-3’
*AtSOC1*-F	5’-CGTAAACTCTTGGGAGAA-3’
*AtSOC1*-R	5’-TCAGAACTTGGGCTACTC-3’
*AtFLC*-F	5’-TTCTCGTCGTCTCCGCCTCC-3’
*AtFLC*-R	5’-TTCCTCCAGTTGAACAAGAGCATC-3’
*AtSVP*-F	5’-CCACATCCACCGACCATC-3’
*AtSVP*-R	5’-AGGACCTTGCCAAGCAGT-3’
*AtMYB33*-F	5’-AGCGGAAACCTCTCTTGGAT-3’
*AtMYB33*-R	5’-TCATTTCCTATGTCATCGCC-3’
*AtFKF*-F	5’-CAGCCATTGGATGATACTTT-3’
*AtFKF*-R	5’-GAGACAACCAGTTTAGAGCC-3’
*AtZAT7*-F	5’-TGTCCGATATGTGGAGTGA-3’
*AtZAT7*-R	5’-TGTAACCAACGAGCCTGA-3’
*AtZAT12*-F	5’-GCGAGTCACAAGAAGCCTAA-3’
*AtZAT12*-R	5’-ATCGGAAACTCCACTCCACAT-3’
*AtHSFA1b*-F	5’-GTTTGGCTCATCATCCTC-3’
*AtHSFA1b*-R	5’-TCCCTCCAGTAACTCATTCA-3’
*AtHSFA2*-F	5’-AAGGCGTTGAACAATCCG-3’
*AtHSFA2*-R	5’-CAGCGAACAACATTTCCATA-3’
*AtRD26*-F	5’-AATGGGTCGTCATCGTCT-3’
*AtRD26*-R	5’-GCATCGTAACCACCGTAA-3’
*AtMYC2*-F	5’-GAACGACCCGTCTATGTGG-3’
*AtMYC2*-R	5’-TTTCGGTTATTGTGCTTGA-3’
*AtMYB96*-F	5’-CTTTGCTTGACCGTTGTT-3’
*AtMYB96*-R	5’-CCTGATGGGTATGGATTA-3’
*AtMYB2*-F	5’-AAAAGCAAGCCAAACACC-3’
*AtMYB2*-R	5’-CACTAATCTCGGCATCCA-3’
*At18SrRNA*-F	5’-CGTCCCTGCCCTTTGTACAC-3’
*At18SrRNA*-R	5’-CGAACACTTCACCGGATCATT-3’

Semi-quantitative RT-PCR was used to analyze the expression of flowering-related genes. Arabidopsis 18S rRNA gene was used as the endogenous control. The PCR products were analyzed through agarose gel electrophoresis and stained with ethidium bromide. All RT-PCR analyses were repeated three times, and one representative was shown.

### Yeast two-hybrid assay

PCR-amplified cDNA fragments of *GmGBP1*, *GmGBP1a* (amino acids 1–189), *GmGBP1b* (amino acids 190–356) and *GmGBP1c* (amino acids 357–612) were cloned into *EcoR*I and *Pst*I site of pBD-*GAL4* as the bait constructs; PCR-amplified cDNA fragments of *GmGAMYB1*, *GmGAMYB1a* (amino acids 1–141) and *GmGAMYB1b* (amino acids 142–538) were cloned into *BamH*I and *Pst*I site of pAD-*GAL4* as the prey constructs. The primers used were: y*GmGBP1*-F and y*GmGBP1*-R; y*GmGBP1*a-F and y*GmGBP1a*-R; y*GmGBP1b*-F and y*GmGBP1b*-R; y*GmGBP1c*-F and y*GmGBP1c*-R; y*GmGAMYB1*-F and y*GmGAMYB1*-R; y*GmGAMYB1a*-F and y*GmGAMYB1a*-R; y*GmGAMYB1b*-F and y*GmGAMYB1b*-R (Table 1). All the bait constructs were transformed into the yeast strain *YRG*-*2* respectively to detect the transcriptional activities.

The yeast two-hybrid assay was performed using the *GAL4* Two-Hybrid phagemid vector System (Stratagene, USA). The yeast strain *YRG*-*2* was transformed with the bait plasmid, and the cells containing the bait constructs that have no self-activation activity were transformed with the prey plasmid according to the manufacturer’s instruction manual. The co-transformants were selected on synthetic complete selection medium lacking Leu, Trp and His. Large yeast clones appearing within 7 days were picked out to test the *LacZ* reporter gene. Positive clones were identified by the restriction digest analyses.

### Gene constructs and Arabidopsis transformation

The *GmGBP1*-overexpression construct was constructed by directionally inserting the full cDNA into the cloned vector pMD18T and then into the binary vector pCAMBIA3300. To make a dsRNAi construct of *GmGBP1*, a 505 bp fragment of *GmGBP1*, 73% of nucleotide homology to AtSKIP, was generated by PCR with primers *GmGBP1*-i-F and *GmGBP1*-i-R (Table 1) and cloned into pJawoh18 vector through attB × attP (BP) recombination cloning. The attB1 and attB2 were the two sequences for the BP recombination reaction (Invitrogen, USA). The constructs were transformed into the *Agrobacterium tumefaciens* strain LBA4404 and used to transform *Arabidopsis* ecotype Columbia using the floral dip method (Clough and Bent 1998).

### Flowering analysis

Seedlings were germinated on MS medium, and then transferred to 1:1 of vermiculite and turfy-soil and grown under LDs or SDs conditions. The flowering time of Arabidopsis was measured by scoring the number of rosette leaves at the flowering time and the number of days from germination to bolting [31]. At least 20 plants were analyzed each time, and the experiments were repeated for three times.

## Abbreviations

ABA: Abscisic acid; GA: Gibberellin; LDs: Long-days condition; SDs: Short-days condition; ROS: Reactive oxygen species; MS: Murashige and skoog; WT: Wild type; SOD: Superoxide dismutase; MDA: Malondialdehyde.

## Competing interests

The authors have declared that no competing interests exist.

## Authors’ contributions

YZ, LZ and WL designed the study. YZ and YG performed the experiments. HL, YL, XW, WT, YH and XZ contributed reagents/materials/analysis tools. YZ, LZ and WL analyzed the data. YZ wrote the manuscript, which was further edited by LZ and WL. All authors read and approved the final manuscript.
